# Psychological Determinants of Investor Motivation in Social Media-Based Crowdfunding Projects: A Systematic Review

**DOI:** 10.3389/fpsyg.2020.588121

**Published:** 2020-12-22

**Authors:** Daniela Popescul, Laura Diana Radu, Vasile Daniel Păvăloaia, Mircea Radu Georgescu

**Affiliations:** Department of Accounting, Business Information Systems and Statistics, Faculty of Economics and Business Administration, “Alexandru Ioan Cuza” University, Iaşi, Romania

**Keywords:** project management, start-up, disruptive innovation, social media, crowdfunding platform, investor motivation, crowdfunding success factors

## Abstract

**Background:** Using the power of Internet, crowdfunding platforms are currently changing the traditional landscape of fundraising. Social media-based IT platforms in particular are bringing the creators of crowdfunding projects closer than ever to potential investors. A large variety of factors function as determinants of individuals' intention to participate in crowdfunding and have an intertwined impact on funding as the ultimate project goal.

**Objectives:** For a better understanding of investor behavior in social media-based crowdfunding projects, this paper covers identifying, analyzing, and classifying general and specific factors of investor motivation, based on the literature in the field.The main focus is the relationship between the affordances provided by social media-based crowdfunding platforms and the psychological determinants of investor motivation in innovative start-up projects.

**Methods:** Using IEEE Explore, Clarivate Web of Science, ScienceDirect, and Scopus, we conducted a systematic review of the existing research on the emerging role of crowdfunding as a disruptive technology in financing the start-up innovative projects. The paper explores the main determinants of investor motivation and aims to streamline the success factors in crowdfunding campaigns.

**Results:** A total of 1,216 publications were identified after searching the aforementioned databases and, upon refining the results, 515 articles were considered for the final sample. After reading the titles and abstracts, the sample was reduced to 78 articles that were read in-depth and synthesized in accordance with the defined research questions. The selected articles were clustered into three main categories: general studies, determinants of investor behavior, and success factors.

**Conclusions:** In the new global economy, crowdfunding platforms have become the nexus between the emerging creators of innovative products and services and the necessary funding sources. This connection is possible via a cumulative collection of contributions from multiple investors recruited from the audience of the selected platform, without time or space constraints. However, the determinants of the investment decision are very different in the case of social media-based crowdfunding platforms compared to determinants in the mainstream environment. This paper surveys these motivators and reveals how platform features can be used to persuade individuals to make a financial contribution toward the success of a project.

## Introduction

There is a growing body of literature that recognizes the importance of crowdfunding, which has emerged as a powerful, popular, and achievable means of funding projects worldwide (Nevin et al., 2017; Rodriguez-Ricardo et al., 2018; Brem et al., 2019; Kim et al., 2020a). The first mentions of the concept in academic literature dates back to 2010, but the number of published articles increased significantly in recent years as a result of the rising interest for using the conjugated power of individuals organized dynamically into “crowds” using technology, as well as due to this financing means becoming legally recognized in more and more countries around the world (Smith and Hong, 2016).

Crowdfunding implies an open call on the Internet, made with the intention of reaching large crowds, in order to get the necessary financial resources to support specific purposes. As a result, relatively small contributions are cumulatively collected from a large pool of people online. The fundraising process is most often called *a campaign*. A campaign can be seen as a project in itself, described as a set of activities with a clearly defined start and end point, geared toward reaching a specific goal—in this case, the goal is to raise the necessary funds in order to carry out the project proposed by the campaign creator (for instance: developing a new product or service). There are three categories of participants in crowdfunding campaigns: (1) the person(s) or the organization requesting funds for a project or a cause (in the case of start-ups, which are the object of this paper, this refers to an entrepreneur), (2) the crowd of potential investors (backers, funders) providing the resources, and optionally (3) the crowdfunding platform. The third element has become increasingly important in recent years: crowdfunding initiatives seem to garner genuine traction via social media-based platforms, by exploiting the truly interactive features that can be “designed and re-designed by humans with relative ease” (Choy and Schlagwein, 2016). Compared to traditional ways of obtaining money, social media-based platforms allow participants to interact with the beneficiaries of the funds via comments, reactions, etc., to follow-up on the status of the crowdfunding campaign and the progress of the funded project. Furthermore, crowdfunding enables reaching out to an unlimited number of geographically dispersed people for the purpose of such a campaign (Mendes-Da-Silva et al., 2019).

Crowdfunding has several unique particularities: it presents a mixture of entrepreneurship with social network participation, in which the customers play an unexpected role as investors; it is time-constrained and involves a variety of roles, including the promoters who disseminate information about the project over social media platforms and the backers who pledge funds for the project (Lu et al., 2014); it has the power to remove barriers to entry (Smith and Hong, 2016); it empowers the users' potential to innovate, as the ideas of many t individuals get support and can be transformed into new products and services (Brem et al., 2019; Jaziri and Miralam, 2019). For these reasons, crowdfunding is more convenient for project creators than mainstream financing channels. Entrepreneurs can present their ideas and plans to a wide audience, in a friendly and interactive environment, and the audience can support the entrepreneurs without requiring them to provide complex business plans and financial indicators that are often difficult to achieve (Wang and Xue, 2019). According to Allison et al. (2015) and Smith and Hong (2016), unlike traditional fundraising methods, crowdfunding has fewer restrictions and a higher financing. Mainstream financing parties, such as banks and venture capitalists, are less interested in backing up start-ups and their projects, which are often in an unpromising embryonic stage. In general, these investors seek projects proposed by mature organizations, with a low level of risk, and have a rather passive attitude. They tend to be interested in the return on investment instead of the product. It is for this reason that crowdfunding is a new and appealing alternative for entrepreneurs, used to generate financial resources without having to call upon traditional sources. Moreover, investors are often potential experts and clients who can support the production and sale process for the products and services proposed by the entrepreneurs. Crowdfunding platforms offer a potentially transformative experience, giving start-ups the possibility to raise funds from a very large number of investors who might become consumers in the future. According to Mollick and Robb (2016), crowdfunding enables the democratizing of financing by eliminating barriers, diminishing restrictions and disseminating innovation. Brem et al. (2019) highlights the impact of crowdfunding platforms at a governmental level, showing that they can be used for the equitable distribution of financing for innovation and thus supporting the underestimated economic power of investor-users.

The benefits of crowdfunding are important in motivating entrepreneurs and do not strictly refer to obtaining assets or financial resources. In the case of start-ups, these can also be substantiated in non-financial benefits, such as attracting employees, engaging the collective intelligence of the crowd, advanced promotion of the products and services or using same as market research and obtaining client feedback, drawing attention from the media, as well as building a pool of future clients. Hu et al. (2015) believe that a crowdfunding campaign can be an efficient marketing and engagement platform for start-ups and for entrepreneurs who wish to promote products that are still unknown. Also, in different types of campaigns, clients are willing to pay for premium access to the product to be developed once the campaign is completed, thus helping to estimate the demand for these new products on the market, as such demand would be difficult to estimate using other methods. Investors get the chance to see companies grow from their incipience, to ascertain if the idea is worthwhile and if a need for radical change becomes apparent throughout the development (De Luca et al., 2019). The chances of products and services being accepted are positively impacted by consumer engagement in the design and development process, and campaign success is an optimal means of highlighting the quality of the project. De Luca et al. (2019) have identified eleven categories of benefits of crowdfunding for entrepreneurs, benefits that are associated with: obtaining financial resources (fund raising and cost management), strategy (business viability and quality of the formulated strategy), marketing (research, client relations, demand), actual operations (product design and development), human resource management (team management), supply chain management (potential partners), and personal aspects (entrepreneurial implications, such as replicating successful experiences, testing communication skills, self-affirmation, boosting confidence and motivation, moral support, etc.). In his turn, Foster (2019) synthesized five reasons why entrepreneurs find crowdfunding attractive: (1) it allows them to finance new projects, keeping their equity capital and avoiding debt; (2) they create a preliminary market, by attracting clients before production is completed; (3) getting their clients engaged in a unique manner, by creating a conversation around the product or service, which can result in obtaining valuable feedback on design and functions, which does not happen in the case of traditional forms of financing; (4) reducing the negative impact of implicit biases associated with underrepresented entrepreneurs; (5) it allows for efficient use of the entrepreneurs' social networks in an inexpensive manner. Crowdfunding is sometimes the only means of financing start-ups, given that mainstream sponsors such as banks and venture capitalists generally seek projects that are more mature and entail lower levels of risk, and are seldom willing to give a change to inexperienced entrepreneurs or to products having uncertain chances of success (Song et al., 2019), particularly given that start-ups financed via crowdfunding platforms are often underdeveloped at the time of their initial presentation. The feedback received from investors thus becomes very important and helps creators to adapt their campaign and anticipate any problems, to get to know their clients' preferences and to address the needs of as wide an audience as possible, which could then become loyal customers. From the entrepreneurs' point of view, Ingram et al. (2014) have identified three major characteristics of the best investors. Firstly, they provide a sufficient amount to cover risks and support the development of the business, in terms of number of employees, volume of products or services, or for advertising. Secondly, the investor brings in additional skills, expertise and a professional network. Lastly, the relation between investors and founders is seen as a potential partnership.

For these reasons, this unconventional instrument is associated with the power to eliminate middlemen from the risk capital industry, with an effect similar to the ones produced by Uber in urban transportation or Amazon in retailing (Smith and Hong, 2016), and to metamorphosise the entrepreneur financing ecosystem (Jaziri and Miralam, 2019) from a series of ivory towers often not accessible to those knocking at their gates into a dynamic, reconfigurable and fertile network. All the aforementioned characteristics transform IT crowdfunding into a genuinely disruptive technology, with a great potential to stimulate innovative projects. Crowdfunding mechanisms are legally recognized by the governments of more and more countries, which leads to their rapid development and increase in popularity worldwide, beyond the traditional western space. The development is rapid or promising in countries such as China (Wang and Xue, 2019) or ASEAN-5 (Indonesia, Malaysia, Singapore, Philippines, and Thailand) (Dikaputra et al., 2019), Eastern-European countries such as Poland and Romania (Fanea-Ivanovici and Siemionek-Ruskan, 2019), but still hesitant in African countries (Jaziri and Miralam, 2019).

After an initial success in the artistic field, online crowdfunding addressed entrepreneurial area, in domains such as technology, knowledge-based start-ups and new product development (Hemer, 2011; Rodriguez-Ricardo et al., 2018). Access to crowdfunding through the Internet has paved the way for many innovative products and services, by reducing the funding gap for innovative start-ups—some funded products are Pebble, the first smartwatch, 3D printers, hardware products, video game consoles, etc.

Hervé and Schwienbacher (2018) analyse the innovative potential of the crowd' participation in the product creation process by providing feedback to the entrepreneur. This feedback can take various forms, including providing ideas on the development of the product during and after the campaign, and providing valuable information on the future demand for the new product. Presenting ideas on crowdfunding platforms can be very important for entrepreneurs, not only because they will access the necessary financial resources, but also for the flows of knowledge that can be collected from their project followers. The online crowdfunding platforms support entrepreneurs' innovative ideas by permitting an open dialogue in the platform and the input of diverse knowledge in their projects, original perspectives of interpretation of the problems they face, and various heuristics for finding solutions. The presentation of the project by a group approved by potential consumers and investors in social media allows fruitful conversations and collection of observations, questions and opinions that can act as catalysts for entrepreneurs and lead to the validation of the idea, to its improvement and its transformation from invention in innovation. New knowledge, with the ability to produce changes and to support the entrepreneurs in reaching their goals, is gathered from various actors. In today's world, due to the high level of technological change and complexity, the ability to successfully access and use knowledge-based values from complementary sources is essential, and crowdfunding offers entrepreneurs this opportunity.

On the potential investors' side of the story, as shown in Kuppuswamy and Bayus (2017), Rodriguez-Ricardo et al. (2018), and Allon and Babich (2020), the individual's levels of innovativeness and creativity and the satisfaction to see an idea turned into reality are key determinants to the intention to participate in crowdfunding. Other motivators identified in our analysis are the investors' desire to transfer their prior knowledge, expertise and experience in the project' field (Saxton and Wang, 2014; Dejean, 2019; Kim et al., 2020a), and the positive relationship between entrepreneur and investor, based on perceived sympathy, openness and trustworthiness (Mollick, 2014; Saxton and Wang, 2014; Agrawal et al., 2015; Moritz et al., 2015; Polzin et al., 2018; Foster, 2019; Mendes-Da-Silva et al., 2019; Song et al., 2019).

To attain the desired success, fund requesting parties and crowdfunding platform managers have to have a very clear understanding of the intentions and behavior of potential investors. It is only provided this condition is met that the project presentation will be able to draw sufficient supporters. The success of a crowdfunding project is entirely dependent on the participation of potential sponsors; this is why understanding their financing intentions and motivations is a fundamental objective of this area of research (Wang and Xue, 2019).

This paper sets out to pinpoint the differences between crowdfunding and the traditional financing mechanisms, to identify what the success of crowdfunding campaigns looks like, and particularly to analyse, based on a systematic review of the relevant literature in the field, the determinant factors of potential patrons' decision to invest in start-up projects. Subsequently, of all these factors we would particularly like to highlight the psychological factors (which we deem essential) and how the characteristics of social media platforms can capitalize on and potentiate them. The rest of this paper is organized as follows. We first provide a brief overview of research questions and methods used in Section Methods. Then, Section Results discusses the differences between crowdfunding and traditional fundrising mechanisms, arguments the disruptive character of crowdfunding, and presents exhaustively the determinants of individuals' intention to engage in start-ups' crowdfunding and the success factors of a crowdfunding campaign. Summary of main findings, limitations and conclusions are given in Section Discussion.

## Methods

This research investigates the emerging role of crowdfunding as a disruptive technology by exploring, among other aspects, the primary motivations of investor in crowdfunding projects. The paper attempts to answer the following four research questions:

1. What are the main characteristics for each type of crowdfunding campaign and the most important platforms used to attract investors?
2. Do the crowdfunding campaigns feature disruptive characteristics?
3. What are the investors' psychological motivations involved in crowdfunding campaigns?
4. What are the success factors of social media-based crowdfunding campaign for the start-up projects?

**Figure 1:**
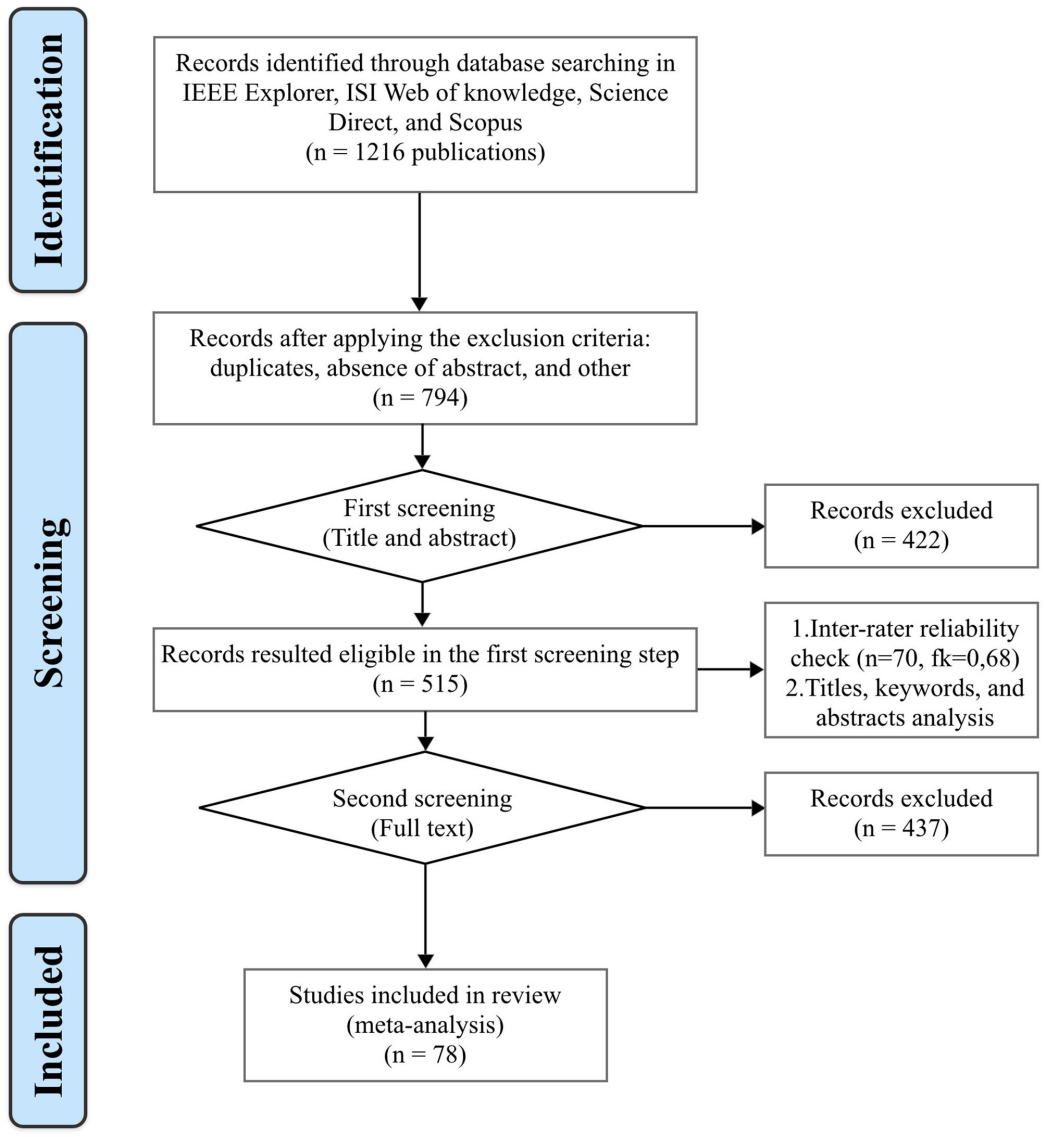
PRISMA 2009 flow diagram. From Moher et al. (2009).

The research follows the design research paradigm presented in Gregor and Hevner (2013). We conducted the research in four major steps (Figure 1). For the first step we performed a comprehensive review of the current literature in the field of crowdfunding. We applied the content analysis technique, “a phase of information-processing in which communications content is transformed, through objective and systematic application of categorization rules, into data that can be summarized and compared” (Kassarjian, 1977). For the first step we defined the filter comprising database, keywords, and type of documents, where possible. We searched using the following electronic libraries: IEEE Explore, Clarivate Analytics Web of Science, Science Direct, and Scopus. We divided keywords into two complementary parts: “crowdfunding” AND (platform OR affordance OR disrupt^*^ OR psychological OR start-up OR motivation) (Tables 1, 2).

**Table 1:** Queries applied for each database.

**Database**	**Search queries**
IEEE explore	[“All Metadata”:crowdfunding AND (“All Metadata”:disrupt^*^ OR “All Metadata”:affordance OR “All Metadata”:platform OR “All Metadata”:start-up OR “All Metadata”:psychological OR “All Metadata”:motivation)]
Clarivate analytics web of science	TS=(crowdfunding) AND [TS=(platform) OR TS=(affordance) OR TS=(disrupt^*^) OR TS=(psychological) OR TS=(start-up) OR TS=(motivation)]
Science direct	(crowdfunding) AND (disruptive OR platform OR affordance OR disruptive OR start-up OR psychological OR psychological OR disruption)
Scopus	TLE-ABS-KEY [crowdfunding AND (platform OR disrupt^*^ OR affordance OR start-up OR psychological OR motivation)] AND [LIMIT-TO (DOCTYPE, “ar”) OR LIMIT-TO (DOCTYPE, “cp”) OR LIMIT-TO (DOCTYPE, “ch”) OR LIMIT-TO (DOCTYPE, “re”)] AND [LIMIT-TO (LANGUAGE, “English”)]

**Table 2:** Search strategy for carrying out the systematic review.

**Search strategy**	**Details**
Keywords	(crowdfunding) AND (disruptive OR platform OR affordance OR disruptive OR start-up OR psychological OR psychological OR disruption)
Databases	IEEE explore, clarivate analytics web of science, science direct and scopus
Inclusion criteria	All papers considered relevant by title, abstract, and keywords
Exclusion criteria	Duplicates, absence of abstract, editorial, letter of editor, opinion, unpublished articles, working papers, and magazine
Period explored	Anytime
Language	English

We tested our queries on a pilot group of articles and we added more keywords if any of the papers in this group was not retrieved by the query string. We restricted the search to articles, literature reviews, chapters and conference papers published in English. The initial group of results was comprised of 1,216 publications, with titles and abstracts related to our research topic. Given the fact that crowdfunding as a research topic is relatively new, we considered all types of scientific publications with no specific time range.

For the following step (i.e., step two) we used Rayyan QCRI^1^ to eliminate redundancies and to extract the scope of the papers.

**Figure 2:**
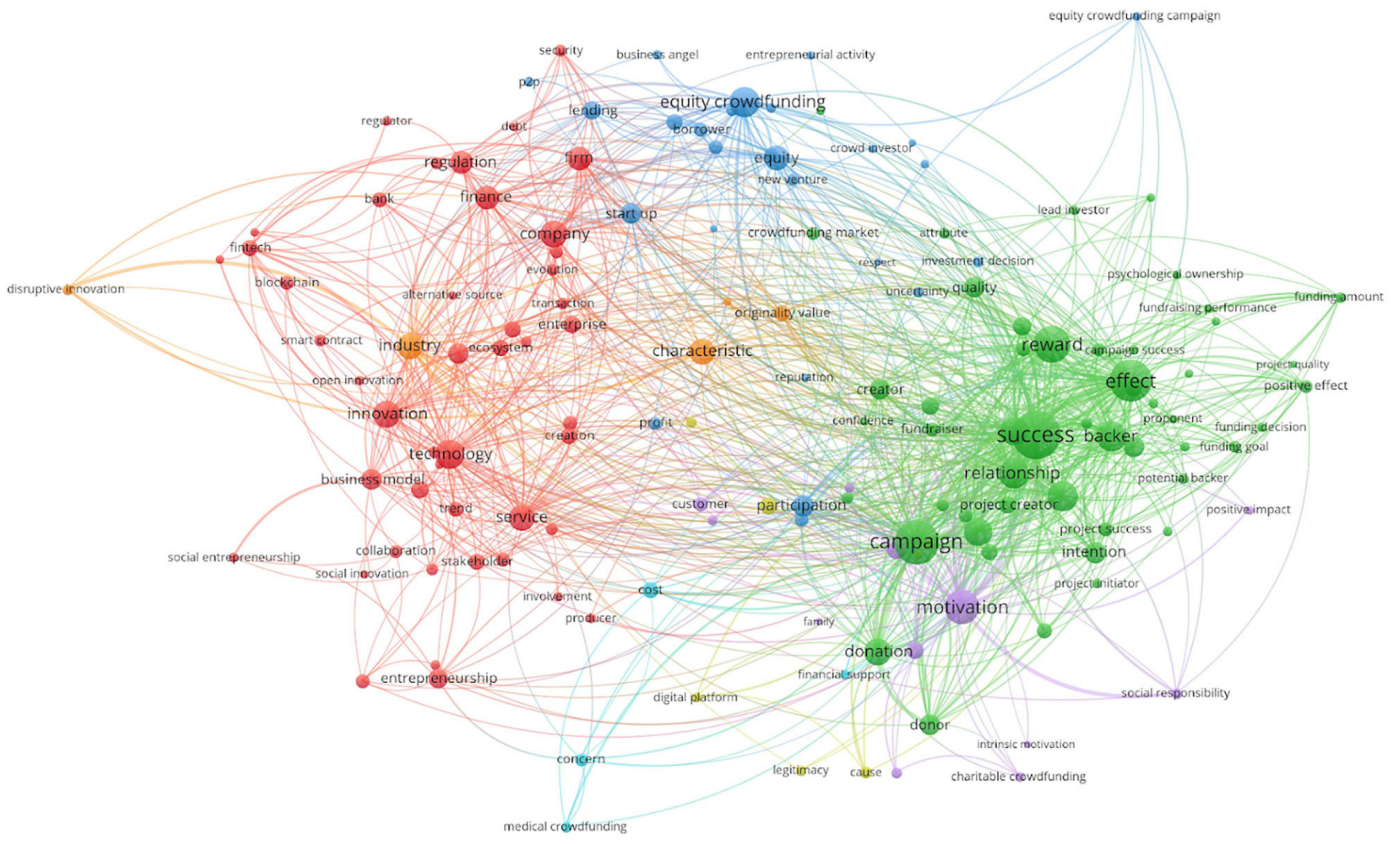
Network analysis of 794 publications using the VOSviewer software.

After the first filtering, we used VOSviewer to identify the main clusters regarding the research topic and to represent the concepts most frequently used in the titles and abstracts of the retrieved papers. VOSviewer is a free software available online at www.vosviewer.com. It allows for bibliometric mapping via identification of keywords determined function of frequency of occurrence thereof and the connection identified between them. The mapping technique is applied to a similarity matrix calculated based on a co-occurrence matrix. The whole network is mapped out in Figure 2. We eliminated general terms, such as article, need, action, case study, etc. In this network, the cycles represent keywords and their diameters indicate the number of occurrences. The distance between keywords reflects the relation between them in terms of co-occurrence links calculated based on the number of publications in which they are used together. Nine clusters were created using the collection of keywords with strong connections.

Based on the keywords from these clusters, in step three we selected articles using Rayyan QCRI. After this selection, the resulting group comprises 515 publications. The check of inter-rater reliability was performed by adapting the procedure used by Mura et al. (2017). In this respect, a sample of 70 articles were randomly selected from the group (*n* = 515) and three of the authors rated them according with a set of five criteria, representative for the selection. Authors judgments were analyzed in SPSS (version 25) by using Fleiss's Kappa statistic. The result (fk = 0.68) returns a good (Laerd Statistics, 2019) level meaning that the strength of agreement between the judgments is acceptable. The result of the inter-rater reliability proved that the selected group of manuscripts (*n* = 515) are consistent with the selection criteria. To identify all relevant research, two review rounds were further performed: first based on title and abstract review and second based on full text eligibility review. The following criteria were applied for papers' abstract: first the article relevance for this study, but also the scientific background, the clarity of the abstract, the objectives of the research, and consideration of the limits of the research. We used these criteria to exclude those papers that are poor-quality or irrelevant for this research. In unclear cases, the decision to exclude or to include an article was made by consensus of all four authors. By applying previous assessment criteria, a large number papers did not meet the inclusion criteria and were therefore excluded.Strict exclusion and inclusion criteria were applied to limit the final selection due to the large number of results obtained throughout the search. All articles focusing on psychological determinants of individual investors in crowdfunding campaigns together with the factors influencing their success in start-up projects were selected. Studies meeting the following criteria were considered for inclusion in the final group: (1) analyzing the psychological determinants or motivations of individual investors in crowdfunding campaigns; (2) investigating crowdfunding campaigns that are dedicated to start-ups; (3) examining success factors influencing crowdfunding; (4) publishing in a peer-reviewed journal or conference proceedings; and (5) availability of full-text article. In this way, the search method that we have applied for the initial set of results ensures that biases and errors are minimized. The above criteria further narrowed down the results to 78 publications that were deemed relevant and reasonable for our research.

In step four, the selected articles were read in-depth and synthesized in accordance with the defined research questions. We recreated the map to analyse and highlight the connections between the analyzed concepts. The five clusters identified by VOSviewer are presented in Table 3 with the following details: color, main and secondary words for each of them.

**Table 3:** Clusters identified via the VOSviewer platform.

**Cluster**	**Main keywords**	**Other keywords**
1 (green)	Crowdfunding	Disruptive innovation, development, innovation, market, value
2 (blue)	Project, platform	Crowdfunding platform, psychological ownership, reward
3 (purple)	Motivation	Startup, success
4 (yellow)	Backer, funding	Crowd, customer, venture
5 (red)	Entrepreneur, investor, campaign	Context, contribution, entrepreneurship, equity crowdfunding, future, investor, knowledge, risk

**Figure 3:**
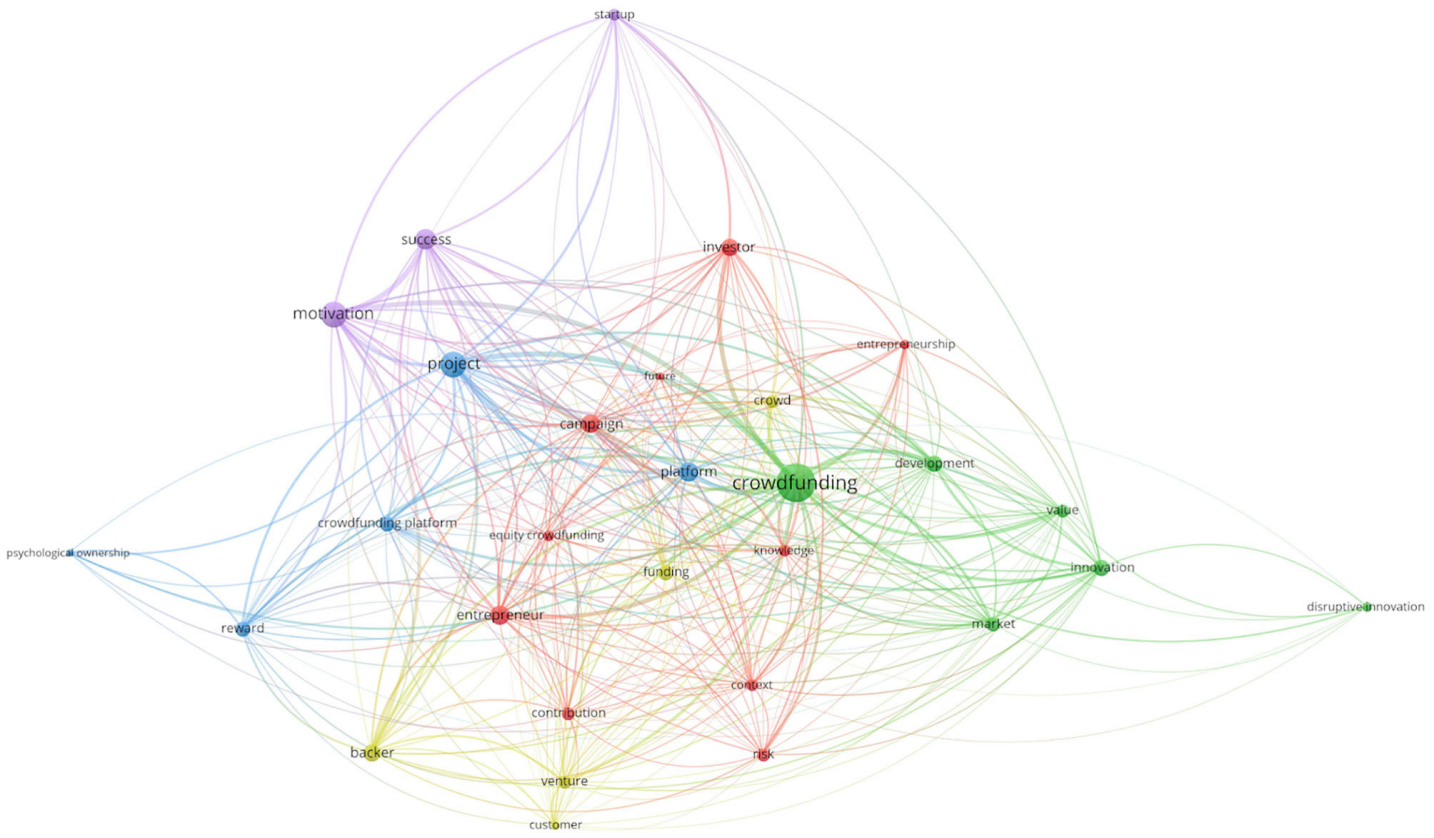
Network analysis of selected publications.

The map obtained via VOSviewer based on the keywords from the selected articles is presented in Figure 3. In this case, we also eliminated the general terms exemplified above.

The selected articles were grouped into three main categories: general studies, determinants of investor behavior, and success factors. Related sub-topics such as benefits, business impact, geographical influences or technology were also deemed of interest for identifying the investors' motivations in relation to crowdfunding. Based on the literature classification, we conducted a research process in order to answer the above-mentioned research questions.

## Results

The research results highlight the existence of a significant difference between crowdfunding and traditional fundraising methods in the context of the disruptive character of modern fundraising mechanisms. To showcase the study results, the following pages also illustrate the essential aspects identified inliterature, presenting determinants of individuals' intention to participate in crowdfunding, as well as the success factors of crowdfunding campaigns.

### Differences Between Crowdfunding and Traditional Fundraising Mechanisms

The taxonomy and examples of crowdfunding platforms that are available for interested parties, as presented down below, help to highlight the differences existing between crowdfunding and the traditional mechanisms used worldwide for the purpose of raising funds.

#### Types of Crowdfunding

**Figure 4:**
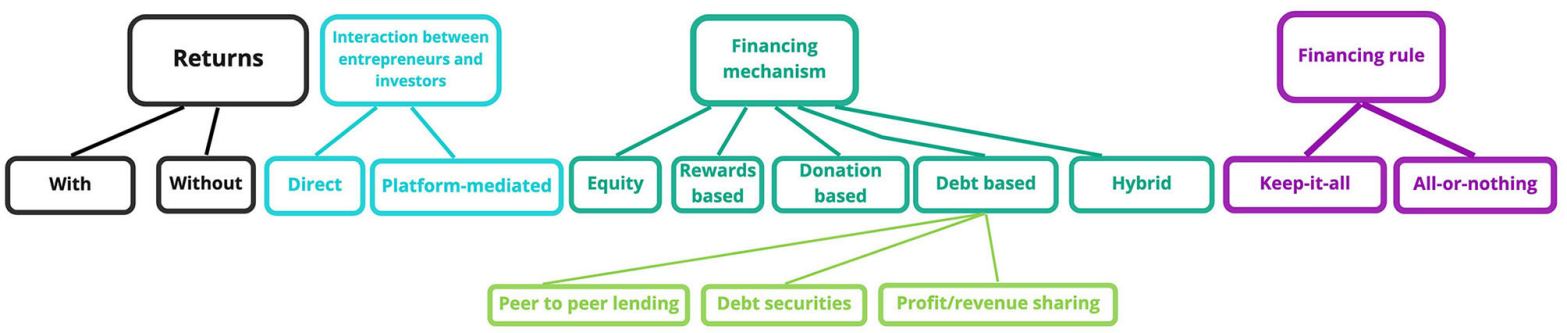
Types of crowdfunding.

To put it most simply, we could break crowdfunding campaigns into campaigns with and without returns (Pichler and Tezza, 2016). Another distinction can be drawn between direct and indirect fundraisers. In the latter case, indirect means that entrepreneurs use crowdfunding platforms instead of directly reaching out to the crowd of potential investors. A study by Mollick (2012) highlights the role of platforms for campaign success, noting that it ensures access to the networks of founders and support for formulating the project specifications. In the absence of platforms, individual entrepreneurs launching their own initiatives should make considerable efforts to activate a network and to highlight the quality of their projects. The main types of crowdfunding campaigns are illustrated graphically in Figure 4.

The types of crowdfunding (Block et al., 2018; Dai and Zhan, 2019; Dikaputra et al., 2019; Dospinescu et al., 2019; Jaziri and Miralam, 2019; European Commission, 2020) are:

- Equity crowdfunding, which entails selling a part of a business to the investors contributing to its growth. The method is similar to trading stocks on the stock market or to a venture capital. Many of the projects in the category of technology start-ups address this type of crowdfunding;
- Rewards-based crowdfunding, where investors expect to receive a non-financial reward in the form of goods or services for their contribution to the project. The typical projects financed using this solution are in the category of games, gadgets, music, and video;
- Donation-based crowdfunding, which entails small donations made by natural persons for the purpose of supporting charitable projects, without expecting a reward in return. Many of the campaigns are geared toward raising funds in order to pay for medical treatments;
- Debt-based crowdfunding, whereby a person or company loans money from a large number of people, undertaking to reimburse the amount within specific time intervals, along with other financial benefits. These are primarily focused on refinancing loans or paying off certain debts generated by the use of credit cards. These can take the following forms:
  ◦ Peer-to-peer lending, a type that is very similar to traditional loans. In this type of crowdfunding, a company loans financial resources from contributors, which the company will reimburse along with a specific interest rate. The large number of investors makes the difference compared to traditional loans;
  ◦ Debt-securities crowdfunding, via which natural persons invest in a debt security issued by the company, such as a bond;
  ◦ Profit-sharing / revenue-sharing, which entails the sharing of future profits or revenues of a company with its current contributors;
- Hybrid models combine different characteristics of the aforementioned types in order to achieve the same goal, i.e., to obtain the necessary resources for financing the projects.

Depending on the financing rule, i.e., the manner in which the entrepreneur receives the resources committed to their project, the literature highlights two common crowdfunding models: the keep-it-all mechanism and the all-or-nothing mechanism. In the former case (keep-it-all model), the entrepreneur receives all the funds that are committed to the project, irrespective of whether the predefined funding goals are achieved or not. In the latter case (the all-or-nothing model), the entrepreneur must collect the amount defined in their funding goal as a minimum, and in case of falling short they receive nothing at all (Foster, 2019).

#### Crowdfunding Platforms

In accordance with the results obtained following the analysis on the specialty literature, the most important platforms used to draw investors are as follows:

**Kickstarter** is the largest online crowdfunding platform in the U.S.A. This platform is a for-profit benefit corporation that considers both the benefits for society and the gaining of profits from its business activities. Kickstarter allows artists and other creatives, as well as companies with new and important products to promote their initiatives via a 30 days' “online campaign,” and to receive financing in the form of “donations” in exchange for rewards, premiums or opportunities to purchase the product as soon as it becomes publicly available. Kickstarter does not sell or retail company stocks, but it does allow start-ups to obtain small amounts as initial financing in order to launch their first batch of products (Smith and Hong, 2016). To this date, 183,274 projects have been successfully financed via this platform. The total dollars pledged amount to $5,050,482,941 and the number of backers adds up to more than 18 million. Approximately 33% of them contributed in financing several projects (Kickstarter, 2020). An interesting feature of the platform is its application of the all-or-nothing rule, whereby the patron's credit card is not charged until the campaign reaches its goal.

**Figure 5:**
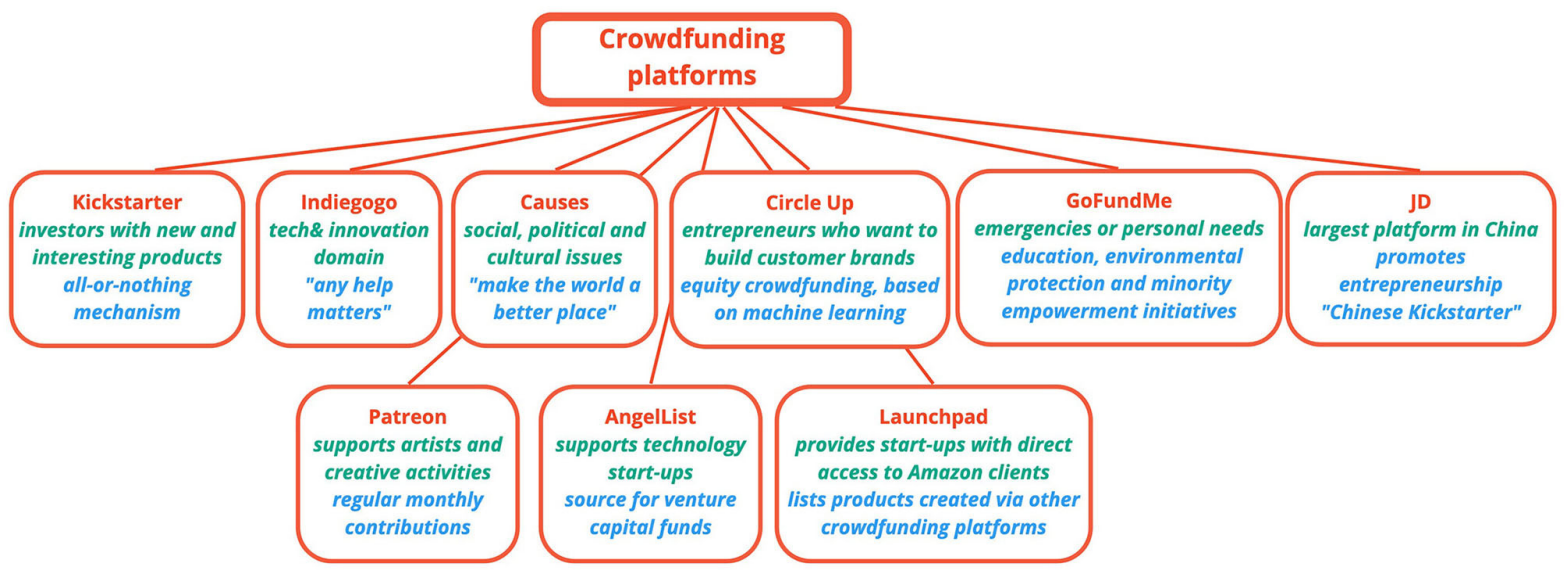
Crowdfunding platforms.

Figure 5 highlights the main crowdfounding platforms used to collect financial resources.

**Indiegogo** is another popular crowdfunding platform in the U.S.A., one of the first of its kind set up for this purpose, more flexible in terms of financing than Kickstarter, as it does not apply the all-or-nothing strategy and believing that any help matters. Approximately 19,000 campaigns are launched every month via this platform, most of these campaigns being from the tech & innovation domain. The platform also has specialists that offer support both for launching and keeping a campaign running, as well as after completing it, i.e., for implementing the proposed project (Indiegogo, 2020). Indiegogo competes directly with Kickstarter and is also present in Canada, U.K., France and Germany (Smith and Hong, 2016).

**Causes** is one of the largest non-profit crowdfunding platform dedicated to fundraising for social, political and cultural issues with a personal or community-level impact for the contributors. It presents itself as *a social network for people who want to make the world a better place* (Causes, 2020). It has more than 186 million registered users in 156 different countries. Both non-profit organizations and individuals can access the platform, raise money for their projects, find people with similar interests, and create petitions for advocacy.

**CircleUp** is an equity crowdfunding platform dedicated to entrepreneurs who want to build customer brands. The platform helped collect more than $390 million for 256 companies and 299 campaigns, but it is more suitable to entrepreneurs wanting to expand their business rather than those who want to launch an idea. The selection process is quite competitive and usually the creator must have a minimum $1 million turnover and growing equity in order for the project to be launched on the website. Helio, a component of the platform, uses machine learning to carry out the strategy of the company asking for help, i.e., it analyses public and private company data from public records, partnerships and information to identify potential investments.

**GoFundMe** is primarily used for emergencies or personal needs, such as education, environmental protection and minority empowerment initiatives (Smith and Hong, 2016), participation in events such as celebrations and graduations, or obtaining funds to finance medical treatments or procedures (~1 out of 3 campaigns are for this purpose).

**Patreon** is particular in that the supporters and donors provide regular monthly contributions to ensure ongoing support for creative activities. It is particularly dedicated to supporting artists. As of its incorporation, the platform drew more than 5 million registered contributors in the following categories video/films, podcast, comedy, comics, games, and education; the contributors support 150,000-plus beneficiaries with more than $1 million.

**AngelList** states that it has invested $ 1 billion in technology start-ups and that several venture capital (VC) funds use AngelList as a sole source of their flow of transactions (AngelList, 2020).

**Launchpad** lists the products created via crowdfunding platforms such as Kickstarter, Indiegogo, Hax and CircleUp. Amazon launched this sales program in July 2015 to help start-ups get their products on the market. This platform provides start-ups with direct access to millions of Amazon clients. Amazon Launchpad is a good example of how established companies can leverage the retailing potential in the early lifecycle of their products (Brem et al., 2019).

**JD** is the most famous and largest reward crowdfunding platform in China, taking up 38.9% of the market share of China's reward crowdfunding market; the crowdfunding platform is also called the Chinese Kickstarter (Wang and Xue, 2019).

#### The Disruptive Character of Crowdfunding

Crowdfunding is a good example of social and collaborative innovation and it has a substantial disruptive potential (Table 4). In the Green Book of Innovation (European Commission, 1995), innovation is regarded as synonymous with the successful manufacture, assimilation and exploitation of novelty features in the economic and social sphere, addressing both the individual needs and the needs of society as a whole. Tapscott and Williams (2010) use the term *social innovation*, referring to a form of innovation present in all the sectors, in which people having motivation, passion and expertise use web-based tools to engage in the endeavor of making the world more prosperous, just and sustainable. Innovation is seen as a state of mind, in which technologies and collaboration are used as catalysts, factors of change with the final goal of ensuring better results for the society. In many cases, these changes are determined by disruptive technologies that permeate societies so deeply that they change their culture and economy. Nowadays, authors consider that the Internet is the most powerful platform that is able to facilitate and accelerate new creative destructions. According to them, “people, knowledge, objects, devices, smart agents converge into many-to-many networks, where new innovations and social trends disseminate at viral speed.” The topic concerning the input of technologies in “generating a new era of prosperity, innovations and collaboration between companies, communities and individuals” is also discussed by Friedman (2007). Referring to *collaborative innovation*, Hislop (2005) and Hurmelinna-Laukkanen (2011) state that the expansion and potential of global networks, the importance of positive network externalities, the need to reach a critical mass of products and the new means of electronic distribution of knowledge have determined companies to accept collaboration in their innovative activities. Nowadays innovation is carried out in a fragmented manner and at a fast-forward speed, obligating innovators to find their place in network-teams with various configurations, capable to quickly respond to challenges.

**Table 4:** The disruptive character of crowdfunding.

**Disruptive innovation characteristics**	**References**	**Why crowdfunding campaigns for startups projects are disruptive in nature?**	**References**
Disruptive innovation creates a new market by providing a different set of values, which ultimately (and unexpectedly) overtakes an existing market. It is often social and collaborative: people with motivation, passion and expertise use web-based tools to get involved in making the world more prosperous and sustainable. Disruptive innovation is open, unpatented–shared innovation is seen as a state of mind that spreads virally.	Hislop, 2005; Tapscott and Williams, 2010; Hurmelinna-Laukkanen, 2011	Crowdfunding emerged as an alternative to the traditional venture capital or to initial public offerings, for the purpose of raising funds with a lower dilution of entrepreneurs' own equity. Start-ups often require direct and quick access to external financing, and crowdfunding has fewer restrictions and a higher financing rate compared with traditional fundraising methods. Crowdfunding evolved in a solid, powerful, popular, and achievable means of funding projects worldwide and is legally recognized in more and more countries around the world.	Cable, 2010; Allison et al., 2015; Oranburg, 2016; Smith and Hong, 2016; Nevin et al., 2017; Rodriguez-Ricardo et al., 2018; Brem et al., 2019; Song et al., 2019; Kim et al., 2020a
		Crowdfunding is social and collaborative: it uses social media-based platforms with truly interactive features. Potential investors and consumers interact with the entrepreneurs via comments, reactions, etc., they follow-up the status of the crowdfunding campaign and the progress of the funded project.	Choy and Schlagwein, 2016
		Crowdfunding empowers the users' potential to innovate, as the ideas of many individuals get support and are transformed into new products and services. It disseminates innovation by creating and using new valuable knowledge through the collaboration between entrepreneurs, investors and final users of a product/service.	Hu et al., 2015; Mollick and Robb, 2016; Brem et al., 2019; Jaziri and Miralam, 2019
Disruptive innovation removes barriers to entry and offers entrepreneurs direct access to the market.	Christensen et al., 2002; Kostoff et al., 2018	Crowdfunding has the power to remove barriers to entry. Entrepreneurs can present their ideas and plans to a wide audience, in a friendly and interactive environment, and the audience can support the entrepreneurs without requiring them to provide complex business plans and financial indicators that are often difficult to achieve. The mechanism support innovative projects, with a high level of risk.	Cable, 2010; Smith and Hong, 2016; Wang and Xue, 2019
		Crowdfunding eliminates the disadvantages of geographical distances between creators and investors. The crowdfunding mechanisms spreads to more and more countries around the world.	Yang et al., 2016; Dikaputra et al., 2019; Fanea-Ivanovici and Siemionek-Ruskan, 2019; Mendes-Da-Silva et al., 2019; Wang and Xue, 2019
		Crowdfunding enables the democratizing of financing by eliminating barriers and diminishing restrictions. It supports the equitable distribution of financing for innovation and the underestimated economic power of investor-users.	Mollick and Robb, 2016; Brem et al., 2019
Disruptive innovation is technology-based, introduces or expands new products/service functionalities, provides products or services with a distinctive structure in terms of costs or prices and allows for the involvement of new consumers/clients in product or service development.	Montgomery et al., 2018	Crowdfunding platforms provide numerous technological advantages such as cost reductions, the access of creators to the resources of investors, efficiency, flexibility and saving the time required for accessing the funds.	Hu et al., 2015; Menon and Malik, 2016; Oranburg, 2016; De Luca et al., 2019; Foster, 2019; Allon and Babich, 2020

Disruptive innovations introduce new technology-based business models that allow the direct access of the population to products and services that would otherwise be too expensive or too complex (Christensen et al., 2002). They can create new markets by appealing to people that previously did not have the necessary resources and skills to get involved in supporting the development of a specific domain. Furthermore, according to Kostoff et al. (2018), disruptive innovations generate growth in the industries they access or the creation of entirely new markets.

Montgomery et al. (2018) have studied the disruptive potential of crowdfunding in real estate projects and identified the following general characteristics of disruptive innovations: they are based on technology, they introduce new functionalities or expand on existing ones, they provide products or services with a distinctive structure in terms of costs or prices, they have limited functionalities and allow for the involvement of new consumers/clients on the market.

Start-ups are innovative projects with high risks, yet significant growth, which often require external financing (Cable, 2010). According to Oranburg (2016), crowdfunding is a newly emerging means of financing start-ups via external investments. It can be very useful primarily for start-ups whose main goal is to produce certain social benefits, as people can be inspired to finance such projects that create public goods. In reality, however, the successful campaigns organized on top platforms such as Kickstarter and Indiegogo are primarily dedicated to developing consumer goods–for instance, one of the most important 15 crowdfunding campaigns was created for such a product (Oranburg, 2016).

With respect to the connection between the characteristics of disruptive innovations identified in the literature and the financing of start-ups, we note that the use of crowdfunding type campaigns also provides numerous technological advantages, the most significant of which are cost reductions, the access of creators to the resources of investors independently of their geographical location, efficiency, flexibility and saving the time required for accessing the funds. This means of financing projects has lower costs compared to classic loans due to the low value of taxes and fees (Menon and Malik, 2016). For investors, the advantage resides in the allocation of small amounts of money, the application of low fees, and the possibility to be directly involved in projects they deem interesting. Moreover, crowdfunding platforms have also simplified both the process of obtaining funds and the process of making investments. All the activities can be carried out online, i.e., signing documents, transfer of funds, monitoring the evolution of the investments, which entails savings in terms of time and financial resources. Furthermore, platforms also provide information to investors, who can carry out analyses on potential investments, of which they can select the ones that best suit their portfolio strategy, risk profile or other criteria. They ensure an audit of the proposed projects before they get posted. However, as these check-ups are not carried out rigorously by all, the reputation of the platform is seen as a major factor for investors in their selection of projects (Wang and Xue, 2019). The benefits of the Internet are undeniable in the case of crowdfunding. It makes it possible for this type of financial involvement to exist in order to support projects and it eliminates the disadvantages of geographical distances between creators and investors (Yang et al., 2016).

Many start-ups that have no access to the other sources of funds, resort to venture capitals for their initial financing. However, venture capital companies or funds reject the majority of proposals advanced to them and only invest in companies that could offer them a high yield on the invested funds. This is why start-ups in several different industries are analyzing crowdfunding as an alternative to the traditional venture capital or to initial public offerings, for the purpose of raising funds in a different manner, with a lower dilution of their own equity (Smith and Hong, 2016).

In the case of start-ups, the amounts offered by venture capital funds substantially exceed the funds obtained via crowdfunding campaigns. One famous example is that of Oculus Rift, a virtual reality headset that obtained $ 2.4 million via Kickstarter and continued to receive $ 75 million worth of financing via venture capital. Subsequently, it was purchased by Facebook for $ 2 billion (Allon and Babich, 2020). As for the entrepreneurs, the success of campaigns brings about the pressure of carrying out their obligations, but what happens in reality after campaigns are over is a topic that requires further research.

### Determinants of Individuals' Intention to Engage in Crowdfunding

Allon and Babich (2020) identify the following motivations of investors: the perspective of financial profit, enjoyment of collaboration (with entrepreneurs or other funders), competition (for instance: gaining advantages for early contributions or access to oversubscribed investments), creation, contribution to a cause (supporting a community cause, such as environmental protection), consumption, charity, sense of belonging, and contract formalization (an official status for the agreement between the entrepreneur and investor) (Table 5).

**Table 5:** Determinants of individuals' intention to engage in crowdfunding.

**Categories**		**Determinants of individuals' intention to engage in crowdfunding**	**References**
Individual motivations	Extrinsic	(Perspective of a) financial profit, material rewards or other similar benefits	Herzenstein et al., 2011; Moritz et al., 2015; Cox et al., 2017; Kuppuswamy and Bayus, 2017; Dai and Zhan, 2019; Dikaputra et al., 2019; Allon and Babich, 2020
		Competition (e.g., obtaining an advantage for early participation)	Allon and Babich, 2020
		Consumption (e.g., priority usage of the funded product/service)	Allon and Babich, 2020
		Quantity and the quality of information provided by the campaigns' creators	Hornuf and Schwienbacher, 2015; Nevin et al., 2017; Wallace et al., 2017; Alcántara-Pilar et al., 2018; Foster, 2019
	Intrinsic	Increasing self-esteem	Estellés-Arolas and González-Ladrón-De-Guevara, 2012
		Creation, innovativeness, the desire to see an idea turned into reality	Kuppuswamy and Bayus, 2017; Rodriguez-Ricardo et al., 2018; Allon and Babich, 2020
		Charitable behavior, altruism	Yang et al., 2016; Cox et al., 2017; Kuppuswamy and Bayus, 2017; Dai and Zhan, 2019; Allon and Babich, 2020
		Development of individual skills	Estellés-Arolas and González-Ladrón-De-Guevara, 2012
	Image enhancement	Prior knowledge, expertise and experience in the project field	Saxton and Wang, 2014; Dejean, 2019; Kim et al., 2020a
Social motivations	Extrinsic	Contract formalization	Foster, 2019; Allon and Babich, 2020
		Cultural factors, cultural differences	Harrison et al., 2010; Lutz et al., 2013; Devos et al., 2015
	Intrinsic	Enjoyment of collaboration (with entrepreneurs/other investors)	Allon and Babich, 2020
		Social recognition or social identification with the crowdfunding community, sense of belonging	Estellés-Arolas and González-Ladrón-De-Guevara, 2012; Nevin et al., 2017; Rodriguez-Ricardo et al., 2018; Dai and Zhan, 2019; Allon and Babich, 2020
		Relationship between entrepreneur and investor (perceived sympathy, openness and trustworthiness)	Mollick, 2014; Saxton and Wang, 2014; Agrawal et al., 2015; Moritz et al., 2015; Polzin et al., 2018; Foster, 2019; Mendes-Da-Silva et al., 2019; Song et al., 2019

*Personal and social traits* are able to increase individuals' intention to participate in crowdfunding, as identified by Rodriguez-Ricardo et al. (2018) in a study on a general audience of potential crowdfunders. *Innovativeness and the social identification with the crowdfunding community have a positive effect on the intention to participate, on attitudes toward helping others and on interpersonal connectivity*, which indirectly determine the intention to contribute to the campaign (mediated by social identification with the crowdfunding community). The investors' perception on the degree of innovation of the presented product, on its quality and its creator's skills determine a positive attitude and involvement in crowdfunding. Wang and Xue (2019) and Choy and Schlagwein (2016) also discuss *individual vs. social motivation*. Nevin et al. (2017) refer to social identity, a person's sense of “who they are,” based on the social group to which they belong.

Studying intrinsic motivation (altruism, the purely internal satisfaction derived from the act of giving), extrinsic motivation (the desire to acquire material rewards or other benefits), and image enhancement motivation, Cox et al. (2017) discovered that *among solely intrinsically motivated funders, those with a desire for image enhancement will contribute with greater monetary amounts to any given campaign compared with funders with no desire for image enhancement*. Another aspect noticed by the researchers refers to the economic profitability of the project as a factor of extrinsic motivation reported to intrinsic motivation. Although rewards are an important incentive (Dikaputra et al., 2019), there is a wide variety of intrinsic incentives that determine individuals to get financially involved in supporting a project, such as *peace of mind, altruism, reciprocity or benefits for the community via implementation of the projects* (Yang et al., 2016; Necula and Strîmbei, 2019). Kuppuswamy and Bayus (2017) highlight the importance of *prosocial behavior* in the case of reward-based crowdfunding: the supporters of the projects wish to make a profit, while also contributing in turning an entrepreneur's idea into reality. In their endeavor to define crowdsourcing, Estellés-Arolas and González-Ladrón-De-Guevara (2012), note the motivation of investors' involvement as a means to satisfy a certain type of need, whether economic or social, such as social recognition, increasing self-esteem, or developing individual skills. Considering the fact that the most popular type of crowdsourcing is crowdfunding, the aforementioned motivations are also applicable in this case. According to Dai and Zhan (2019), the prosocial motivations of consumers that help creators reach their funding goals substantially impact the financing activities on these platforms and can exceed the economic considerations highlighted by previous research. The prosocial motivation is the internal condition that activates, directs and supports the pursuit of goals and increases as their completion date approaches, a phenomenon known as *the goal gradient effect*. The mentioned authors show that drawing closer to the goal has a higher positive effect on the level of support for the project if the project is drawing closer to its financing term, if the target amount is relatively small, or if the project has limited early support. As a result, aside from the declared benefits, in the case of reward-based crowdfunding, investors also have intrinsic motivations. They wish to feel that individual contributions have a positive impact on the project, which determines them to engage in prosocial behaviors.

Regarding the relationship with the project creator, Polzin et al. (2018) distinguished between in-crowd and out-crowd funders (funders with and without ties to project creators) and discovered that *in-crowd investors rely more on information about the project creator than out-crowd investors*. For financial return crowdfunding, financial information becomes less important once a strong relationship is established with the project creator. The advice for project creators is to target information to specific audiences based on their relationship strength across different types of crowdfunding projects. Also, Moritz et al. (2015) signaled that *perceived sympathy, openness and trustworthiness in the relationship between entrepreneur and investor* is of significant importance. Mendes-Da-Silva et al. (2019) identify the entrepreneur's network of close contacts as a factor that might play a central role in funding. In the same vein, Mollick (2014) shows that personal networks are associated with the success of crowdfunding efforts. The entrepreneur's position in their social network works as an indicator for the success or failure of the proposed project (Foster, 2019). Agrawal et al. (2015) notice that in the first phases of a crowdfunding campaign, family members and friends are important patrons. In the first phases, social networks can contribute toward improving the reputation of start-ups and can operate as a signal in respect of their quality (Song et al., 2019). Consequently, for the success of start-ups, developing the social networks of entrepreneurs is an essential prerequisite. However, additional knowledge is also necessary concerning consumer marketing and social networks to ensure the boosting of communication efficiency. On the other hand, the lack of entrepreneur preparedness makes it necessary to provide detailed information about the product, which increases the vulnerability in terms of intellectual property right theft. Foster recommends that entrepreneurs should not rely exclusively on a high level of support from the strong connections in their social network, and that instead they should aggressively promote their project to as many audiences as possible (Foster, 2019).

The *quantity and the quality of information* needed to allow entrepreneurs and potential investors to be interested in each other is also a key point of the process. This aspect is even more important in the early stage when poor information is available and information reliability is not very clear. Hornuf and Schwienbacher and also Foster find that specific kinds of information, such as *updates to investors*, significantly drive investment as funders update their preferences in light of the project assessment (Hornuf and Schwienbacher, 2015; Alcántara-Pilar et al., 2018; Foster, 2019). Information asymmetry, along with the heterogeneity of participants in crowdfunding campaigns, at the level of both patrons and entrepreneurs, as well as the control mechanisms of specialized platforms, impact the result of the projects. This especially refers to the differences between the information held by the two parties, caused by contradicting interests between the potential entrepreneur and investors. Usually, investors do not possess the skills required for assessing the projects and technologies proposed by the entrepreneur, while entrepreneurs tend to be reluctant about revealing all the information about the proposed products/technologies and their potential on the market. Consequently, it is difficult for investors to identify the information they need to assess the quality of start-ups and to distinguish between promising and unprofitable investments (Meoli et al., 2019). The matter of asymmetry is very important in crowdfunding campaigns that are carried out online for a short period of time. For instance, due to information asymmetry, non-profit projects that have a lower variation of value are more likely to obtain more financing (Moritz et al., 2015). Potential entrepreneurs could highlight the quality of projects by showcasing certain features thereof in order to help overcome the uncertainty and information asymmetry and to grant credibility to the project. Social networks support the flow of information signaling the quality of the projects and entrepreneurs (Polzin et al., 2018). Being more active on social media and having a higher level of engagement with the crowd will have a positive impact on the overall funding of a crowdfunding campaign (Nevin et al., 2017). Charities and non-profit organizations recognize the value of online social media platforms for influencing consumer responses, particularly among younger consumers (Wallace et al., 2017).

Another category of studies analyzed the financing decisions and/or the behavior of patrons in the traditional environment compared to those involved in crowdfunding. Unlike professional investors, those who support projects in crowdfunding campaigns are substantially influenced by non-standard information, as well as by the status of the campaign. For instance, projects with multiple “backers” or that closer to being successfully financed can draw more potential investors, a phenomenon known as the “herding effect” (Herzenstein et al., 2011). Their study was conducted based on information sourced from prosper.com. In one of their previous studies, they also discovered that information *that cannot be verified affect the investors' decisions more than objective and verifiable information*.

*Trust, knowledge in the domain of the project, expertise and experience* could be also factors of the crowdfunding decision. Saxton and Wang (2014) consider that the *presence of trust* is fundamental for crowdfunding and has a direct impact on the intention to invest. To gain the investors' trust, entrepreneurs have to provide accurate and complete information to potential investors from the very start. The level of trust indicates the extent to which investors believe the start-up has the capacity to succeed and to achieve the desired results. Moreover, a high level of trust also boosts the clients' willingness to share the necessary personal information to purchase the products or services that will be supplied by the company and correlates positively with consumer loyalty. According to Kim et al. (2020a), the extrinsic and intrinsic motivation impacts the level of trust in the project. Although crowdfunding platforms provide various means to collect information about the project founder, such as the founder's previous experience, motivations and details about the project, tacit knowledge remains important in establishing trust and mutual commitment (Dejean, 2019).

In regards to *cultural differences* on crowdfunding dynamics, some studies draw on the cultural entrepreneurship literature to assess whether a borrower's cultural alignment with their own country increases or decreases funding speed (Harrison et al., 2010; Lutz et al., 2013; Devos et al., 2015).

### Success Factors in Crowdfunding Campaigns

The success of a crowdfunding project/campaign can be reflected in the performance of targeted fundraising. When the amount of funds collected via a crowdfunding project/campaign exceeds the target amount, then the crowdfunding project can be deemed successful (Dikaputra et al., 2019).

Chen et al. (2020) classify the factors influencing the success of crowdfunding campaigns into two categories, i.e., static factors and dynamic factors, with different effects function of the campaign phase. Static factors refer to those elements that do not change during the fundraising campaign, such as the entrepreneurs' social capital, information about the projects' description and the funding goals. Dynamic factors, on the other hand, change as the fundraising process progresses. These factors refer to financing performance, project popularity and the public's reactions to the entrepreneurs' attitude.

Previous studies show that, although there is quite a large number of successfully financed campaigns, many of them fail to achieve their ultimate goal. For instance, according to the statistics provided by Kickstarter, only 37.81% of the projects promoted via this platform met their target goal (Kickstarter, 2020). Success is influenced by factors such as the duration of the campaign (Mollick, 2014), the financing project (Muller et al., 2013), certain expressions, readability and length of description, project advertising mode (text, photos, video, etc.) (Greenberg and Gerber, 2014; Dey et al., 2017), frequency of information updates, existence and level of rewards (Greenberg and Gerber, 2014) and the number of shares on social media platforms (Kaartemo, 2017). The factors that influence the evolution of the campaign within social media networks are both qualitative (for instance: video footage/animated images) and quantitative (the size of the entrepreneur's social network, the number of comments or number of updates).

Yang et al. (2016) identified two essential components for the success of crowdfunding campaigns, which are directly connected to entrepreneurs. First of all, the entrepreneurs' social network plays an important part, and it should be as sparse as possible in order to be more efficient. Second of all, the entrepreneurs' experience and the decisions they make during the project are an important factor. Studies show that the entrepreneurs that have previously run successful projects have higher chances of obtaining new financing (Koning and Model, 2014). Also, providing detailed information about the project, the implementation plans and associated risks have a favorable influence on the chance to obtain financial resources (Ahlers et al., 2015). Chen et al. (2020) classified the success factors in two cathegories, static and dynamic. Table 6 aim to complete the above mentioned classification with other factors as they have been identified in the literature.

**Table 6:** Success factors in crowdfunding campaigns.

**Category**	**Success factors**	**References**
Static	Entrepreneurs' social capital	Chen et al., 2020
	Entrepreneurs' funding goals	Chen et al., 2020
	Existence and level of rewards, including prior financing received by the entrepreneur or the existence of multiple financing parties (venture capitalists or business angels)	Greenberg and Gerber, 2014
	Entrepreneurs' previous experience in the project field	Koning and Model, 2014; Yang et al., 2016; Kleinert et al., 2018
	Size of the project	Dikaputra et al., 2019; Xie et al., 2019
	Certain characteristics of the individuals seeking to raise funding: gender, location of the initiator(s) (peripheral geographic areas, proximity), team size	Agrawal et al., 2015; Moleskis et al., 2018; Dejean, 2019
		Greenberg and Mollick, 2017; Moleskis et al., 2018; Sauermann et al., 2019
	Risks associated to the project	Ahlers et al., 2015; Moleskis et al., 2018
	Quality of the implementation plan	Ahlers et al., 2015
	Platform features (reputation, trust, webpage visual design)	Dai and Zhan, 2019; Wang and Xue, 2019; Kim et al., 2020b; San Martín et al., 2020;
	The presentation of the project: images, video footage/animated images, text—use of certain expressions, readability and length of description	Muller et al., 2013; Greenberg and Gerber, 2014; Mollick, 2014; Ahlers et al., 2015; Dey et al., 2017; Koch and Siering, 2019; Moradi and Dass, 2019; Sauermann et al., 2019; Chen et al., 2020
	Emotional appeal, signaled popularity	Koch and Siering, 2019
Dynamic	Financing performance	Xie et al., 2019; Chen et al., 2020
	The public's reactions to the campaign and entrepreneurs' attitude	Koch and Siering, 2019; Chen et al., 2020
	Number of investors (goal gradient, herding effect)	Xie et al., 2019
	Duration of the campaign	Mollick, 2014
	Frequency and quality of information updates	Greenberg and Gerber, 2014; Xu et al., 2014; Ahlers et al., 2015; Block et al., 2018; Hornuf and Schwienbacher, 2018; Foster, 2019; Koch and Siering, 2019
	The popularity on social media platforms of the project and of entrepreneur (including advertising by photos, video, text, the size of the entrepreneurs' social network)	Lu et al., 2014; Mollick, 2014; Yang et al., 2016; Butticè et al., 2017; Kaartemo, 2017; Dikaputra et al., 2019; Sauermann et al., 2019; Xie et al., 2019; Yeh et al., 2019; Chen et al., 2020
	Entrepreneurs' decisions during the project	Koning and Model, 2014
	The social ties between geographical regions	Dejean, 2019
	Patents	Meoli et al., 2019

According to Xu et al. (2014), a campaign that features frequent updates with progress report information, newly added contents, answers to questions, added rewards, etc. has 26% higher chances to succeed than a similar campaign with information that is not updated. Moreover, there are some platforms (such as Kickstarter) that rank the projects on their page based on their popularity. Block et al. (2018) discovered that posting an update has a significant positive effect on the number of investments made by the crowd and the collected amount. The effect is not entirely immediate, but rather gains traction a few days after publishing the update. Furthermore, the effect of the updates loses its statistical significance once there is an increase in the number of updates posted during a campaign. Using plain and clear language in the updates boosts crowd participation, while the length of the update (number of characters) has no effect (Mitra and Gilbert, 2014). As for the contents of an update, we discover that the positive effect can be attributed to updates about new developments regarding the start-up, such as new financing, business developments and cooperation projects. Updates on the initial team, business mode, product evolution and advertising campaigns do not have significant effects. Updates allow start-ups to signal their value to the crowd and to establish their credibility and legitimacy during a crowdfunding campaign. Consequently, creators should generate daily traffic on the webpage of the project, which can be achieved by frequently updating the provided information. A study conducted by Moradi and Dass (2019) shows that crowdfunding campaign creators should use negative framing, such as using counterfactual language to highlight the costs associated with the lack of contribution in the description of their project, as this type of framing has a positive impact on the level of financing. Other similar elements with a positive effect on the favorable decision of investors are brief text updates, the presence of a link to a website where the project is presented, the presence of comments (Dikaputra et al., 2019).

Success is related to certain characteristics of the individuals seeking to raise funding. For instance, studies suggest that campaigns launched by women or by teams that include at least one woman have greater chances of success than campaigns launched by men or be male-only teams (Greenberg and Mollick, 2017; Sauermann et al., 2019). Other essential elements are the social interconnection of the creator via social media networks (Mollick, 2014; Butticè et al., 2017) or campaigns launched by persons located in peripheral geographic areas (Agrawal et al., 2015).

A study by Dikaputra et al. (2019) shows that in ASEAN-5 countries, small-sized projects are more likely to be funded; also, potential backers prefer large teams, which is consistent with the resource-based view of firms.

The success of projects is also influenced by the following factors: project characteristics—projects developed for non-profit purposes are more likely to be funded than projects created for-profit; budget–projects with small budgets have higher chances of reaching their goals; as well as the radical and innovative character of the projects, which substantially contributes to the chances of success. As far as the connection between the success of a crowdfunding campaign and its characteristics is concerned, the presented information, the presentation manner and how creators interact with the crowd are all essential. Researchers have established the following positive correlations: the quantity of information provided about a project correlates positively with the success of funding, the information provided in a visual form, including via videos, are particularly useful, frequent project information updates during the campaign can further increase the likelihood of success, support from a business angel or venture capitalist correlates positively with the success of the fundraising, campaign anticipation by providing information about the project via social media networks (Sauermann et al., 2019).

Another study conducted by Kleinert et al. (2018) shows that in the case of financing start-ups via equity crowdfunding-type campaigns, the existence of prior financing received by the entrepreneur from venture capitalists or business angels gives greater chances of success compared to projects that have not received such financing. Also, the existence of multiple financing parties has a stronger favorable effect than when there is a single financing party, and the existence of previous successfully completed campaigns has a positive influence both on the number of investors and on the probability of financing, particularly in the case of companies with low human and social capital, as well as in initial phases when uncertainty is very high.

In a study on Taiwan and Japan, Yeh et al. (2019) developed a useful framework comprising two aspects and four factors that support crowdfunding success. Specifically, they highlight the aspects of attraction-promotion and cognition-promotion and the factors of media richness, attention, signaling and kindness. The authors analyse how the aforementioned factors influence the success of crowdfunding so that founders can use these factors to obtain the funding they need and increase the probability of success for their project. In a study on a Chinese crowdfunding platform (taobao.com), Xie et al. (2019) also used a set of five variables (detailed below) to perform a regression analysis so that the effect of each variable can be quantified. They discovered a significant positive relationship between the funding amount and funding target, as well as the number of investors and number of followers having a positive effect on the funding amount. The results of the statistical analysis identified the above as the most influential variables for funding success in four project categories: (1) science and technology, (2) entertainment, video, design, and animation, (3) agriculture and donation, and (4) games and books. In their research, Leone and Schiavone (2019) determine that the success of crowdfunding can result from a greater adoption of the founders' social capital size, and from other post-failure revisions (e.g., product redesign, different funding period).

Koch and Siering (2019) find that emotional appeal has a positive impact on successful project funding, along with signaled experience or popularity. They also show that both information and risk disclosure have a positive influence on funding success. As such, a higher amount of information transmitted through text messages, pictures, and videos reduces uncertainty regarding the project and diminishes the investors' resistance and hesitation. At the same time, they proved that too much information harms the funding process.

Moleskis et al. (2018) perform an econometric analysis investigating how the three success factors (risk, lender proximity, and gender) impact the nature of the project. They analyzed humanitarian and entrepreneurial projects.

Underlying project quality is identified by Mollick (2014) as an important determinant of success in crowdfunding. The temporal distribution of customer interest in regards to a project is reciprocally affected by both the freshness and the remaining duration of the project. The results of a project are more deeply correlated with the early promotional activities on social media rather than its own properties. A project is popularized via massive promotion, whereas the keystone of its success is established in the intensive interactions between participants (Lu et al., 2014).

**Platform features** are important and can potentiate the other factors. It has been demonstrated that the number of likes, shares, favorites, retweets, the number of posts, the quality of posts, response speed, engagement in a campaign influence its success. Investment decisions are rooted in such collective network interactions (Hornuf and Schwienbacher, 2018). Of the more special features that help boost the level of investor engagement we would note the *stretch goals* feature, which allows projects that have already been successfully financed to continue the campaign and to up the *ante, offering extra products or additional features for investors that wish to continue to participate* (Foster, 2019). Furthermore, Foster (2019) also shows that the platform helps potential investors to assess the support provided by their social network and thus reduce the information asymmetry. The results of a study conducted by Kim et al. (2020b) show that the willingness to get involved in a crowdfunding campaign is influenced to a larger extent by the trust in the platform than in the fundraising party. The platform perceived risk is also postulated by San Martín et al. (2020) as capable of influencing individuals' attitudes toward and intention to participate in a crowdfunding project. Crowdfunding platform reputation and webpage visual design are also identified by Wang and Xue (2019) as major factors in making the decision to invest in a campaign. Dai and Zhan (2019) advise crowdfunding platform managers to consider the sponsors' prosocial motives when designing platform functionalities. To attract sponsors that are willing to have an impact on projects that are close to their funding goals, they could consider activating an advanced search option by goal proximity. Moreover, from a project planning perspective, crowdfunding platforms should pay attention to the progress of all the projects in the same category and dynamically decide when to launch new projects, in order to reduce competition between projects that are close to reaching their funding goals and new projects.

In the case of equity and debt crowdfunding, which are subject to stricter legal regulations, platforms are on the one hand making the relations between supporters and entrepreneurs official, replacing the informal family and friendship ties (Agrawal et al., 2014), and on the other hand they can be used as an alternative retail channel (Allon and Babich, 2020).

*The cost of distance in the geographical flow of crowdfunding cannot be neglected*. In fact, most metropolitan regions shape the geography of funding. According to Dejean (2019), the social ties between regions are one of the important factors in determining the flow of funding. However, could the number of immigrants in a region or labor mobility increase the crowdfunding flow, or does the elasticity of distance remain important and do social ties between regions determine the flow of funding? By means of social networks, we appreciate that it is possible to mitigate this tendency.

Meoli et al. (2019) *have studied the role of patents in the attraction of investors in reward-based crowdfunding*. Unlike professional investors, such as venture capitalists, for whom holding a patent for the product to be developed via the start-up is a favorable argument for financially supporting the project, in the case of backers the authors identified that this aspect correlates negatively. One reason could be the association of patents with a higher technical complexity, which causes individuals to perceive patent-based projects as more high-risk, less familiar in terms of the scope of use, and less engaged in social causes. Moreover, they signal a high level of innovation, which causes them to be perceived as very removed from the market and less usable by the general public.

## Discussion

The systematic analysis carried out in this paper revealed a large body of literature produced on the topic of crowdfunding, with an emphasis on its role as a feasible means of funding projects around the world.

### Summary of Main Findings

The study achieved its objectives by producing several key findings.

1. The literature analysis has shown that crowdfunding is considered a social and collaborative innovation platform that proves to comprise a considerable disruptive potential. As with other disruptive innovations, crowdfunding introduces new business models that are technology-related and facilitate its use on a less complex and less expensive basis. Moreover, crowdfunding as a disruptive innovation has the following main features: it relies on technology, it adds new functionalities or builds on existing functionalities, it provides products or services with a distinctive structure in terms of costs and that are conducive to involving new consumers in the market.
2. Consistent with the literature, this study found a large variety of factors among the determinants of individuals' intention to participate in crowdfunding projects. Personal and social traits appear to boost the individuals' intention to engage in crowdfunding. Another set of factors include the intrinsic (altruism, the internal satisfaction derived from the act of giving), extrinsic (the desire to reap material rewards or other benefits), and image enhancement motivations. In this respect, among intrinsically motivated funders, the study shows that those with a desire for image enhancement are stimulated to increase their financial contribution to any given campaign compared to those with no interest for image enhancement. Consequently, the economic profitability of the project as an extrinsic motivation factor, in relation to the intrinsic motivation is also very common among the factors. Although rewards are an important incentive, there is a wide range of intrinsic incentives such as gratitude, altruism, reciprocity, or community benefits through project implementation that stimulate funders to become involved financially to support a project.
3. This study confirms that the factors influencing the success of crowdfunding campaigns are divided into two categories, namely static and dynamic factors. While static factors do not change during the campaign (such as the entrepreneurs' capital, project description, and funding targets), dynamic factors change as the crowdfunding process progresses. The latter category of factors refers to funding performance, project popularity and public reaction to the attitude of entrepreneurs. Another set of factors for the success of crowdfunding campaigns include the social network, the expertise and experience, and the decisions made by entrepreneurs during projects. Studies show that entrepreneurs that have previously carried out successful projects and have a sparse social network are more likely to obtain new funding. Also, providing detailed information about the project, implementation plans and associated risks favorably influences the chance to obtain financial resources. Other success factors listed in the literature review are certain characteristics of the individuals seeking to raise funds (the inclusion of women in the crowdfunding project team), social interconnection (of the creator through social networks), project size (smaller projects are more likely to be funded) and the size of the team (potential backers seem to prefer large teams).

**Figure 6:**
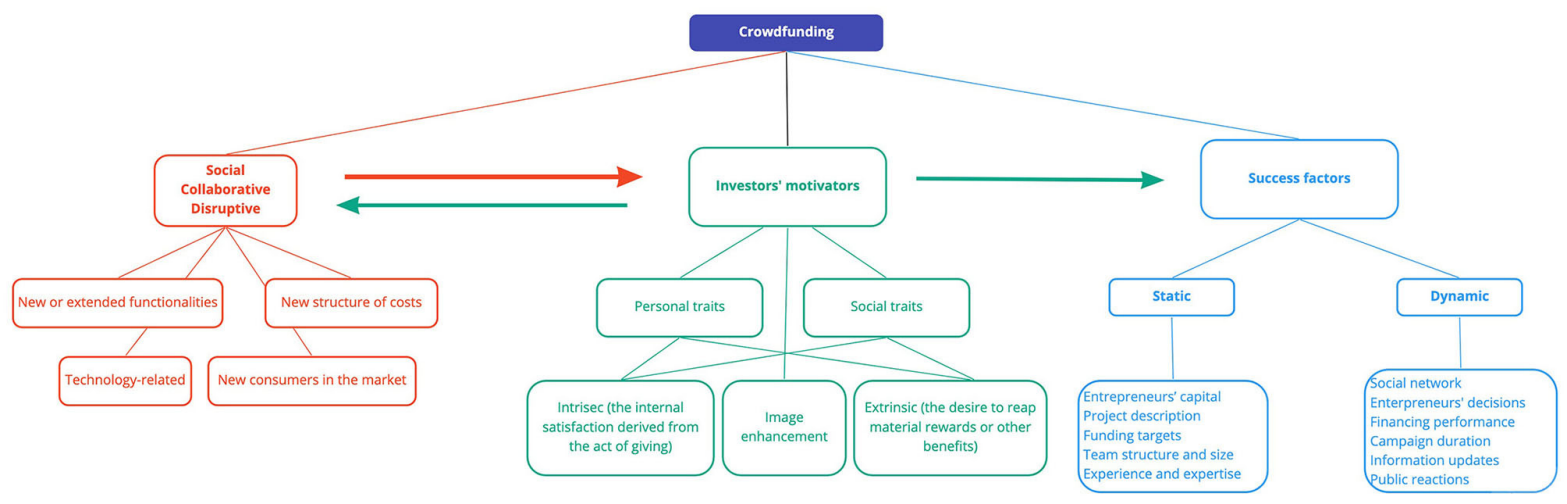
The main findings of the study.

The main findings of the research are summarized in Figure 6.

Besides the theoretical contribution, the study also has practical implications, being of interest for the individuals/companies currently using or expecting to use social media-based crowdfunding campaigns in order to finance their innovative start-up projects in different parts of the world. In our opinion, there are two main categories of beneficiaries that could concretely fructify the results of this research. (1) For entrepreneurs interested in launching a start-up, detailed knowledge of the factors that motivate the members of the “crowd” to invest is useful in building the fundraising campaign in such a way as to achieve the desired result. The chances of success for the campaign and the project proposed by the entrepreneurs increase due to the correct choice of the platform (1), of the target group (2), by pertinent decisions on the included content and on the manner of presenting them (3) and by constructing messages that take into account the stimulation of many of the determinants identified by us above (4). In addition, our study reveals that the triggers of the funding decision differ from one stage of the funding campaign to another, that the types of funders (in-crowd, out-crowd) values different information, and, in addition, the success of a campaign also depends on how it allows the ICT platform to be built, the size of the project, etc. (2) For managers and designers of crowdfunding platforms, the study is of interest because it highlights the characteristics of the platforms that influence the success of the projects they present. The synthesized results can be used in the design of key functionalities, interface, platform usage scenarios, so as to stimulate investor participation, achieve the goal of entrepreneurs and foster innovation in the domain.

The study streamlines that understanding the crowdfunding financing mechanism (which proves to be more flexible compared to traditional mechanisms), the determinants of the decision to invest and the success factors can be very useful for entrepreneurs in turbulence periods such as the one generated by SARSCov2. The above pieces of information are forming a safety net aiming to transform innovative ideas into reality.

### Limitations

This article is not lacking in limitations. The first limitation is the strictly theoretical character and the lack of empirical testing. Future research should focus on the empirical evaluation of psychological motivations underlying the engagement of individuals in supporting start-ups financially via crowdfunding-type campaigns. Another aspect is the fact that entrepreneurs aim to develop and supply highly varied products and services upon initiating a start-up. Future research should expand on and particularize the determinants identified in specific fields of activity. Even though there are certain limitations mentioned in regards to them, the motivations analyzed in this paper can be used to boost the chances of success for crowdfunding-type campaign creators. Similarly, they could use the success factors identified in this paper to coordinate their projects in such a manner as to increase their chances to collect the necessary financial resources. Furthermore, a limitation of this study is that the subject of crowdfunding is very topical, technology-driven, and therefore very dynamic. More papers are being published every day and the current results are subject to new amendments brought about by new technology advancement and legislative changes.

### Conclusions

In principle, start-ups are innovative, often niche-type projects, that nevertheless engender high risks, but also a significant potential growth and that oftentimes require external financing which is quite difficult to obtain. The financing manner, the relevant competition, and the types of financed projects have evolved and increasingly migrated online, which further complicated the equation for selection, particularly due to the psychological determinants of investor motivation.

Following the systematic literature analysis carried out in this research endeavor, we noted that in comparison with traditional project financing mechanisms, crowdfunding platforms democratize the access to funds, giving a significant chance to start-ups that generate innovative—but often risk-prone—ideas. Thus, while traditional project funding usually entails submitting complex documentations in the context of a competition restricted by the formalities of professional language, focused on proving one's eligibility and the financial capacity of the applicant requesting the funds, followed by the analysis of the received proposals and offering an answer after a usually long period, crowdfunding platforms allow entrepreneurs to present their ideas to a very large mass of potential investors as soon as they deem themselves ready, pointing out the information they deem essential in a brief and dynamic presentation that can be updated instantly function of the feedback it elicits. The social and collaborative potential of crowdfunding platforms provides investors with significant further benefits aside from attaining the desired financial goal. As early as the phase preceding the conception of the project proposed for financing, by leveraging the advantage of direct communication via the platform, investors can contribute in the design of the product/service, in configuring a market for the latter, thus reducing the level of risk associated with each innovative idea.

However, the financing party—an individual in a crowd of individuals—is structurally and deeply different from traditional financers. Some factors that also matter in their decision to invest also include: the perception on the degree of innovation/quality of the proposed product or service, identifying with the entrepreneur and confidence in their skills, their proposed project and/or the community of financers, the benefits in terms of reputation, pragmatic rewards, as well as very strong elements of intrinsic motivation: personal satisfaction, altruism, reciprocity or the benefits to the community via the implementation of projects. Unlike traditional financing, what really matters in crowdfunding is the relationship that is created or improved between the entrepreneur and the potential investor. A mutual liking for one another, transparency, and the capacity to generate trust are essential when the project creator is unknown to the potential investor. The size and quality of the entrepreneurs' network are also essential elements for influencing investor behavior. Trust, as an essential attribute of the relationship between the two parties, is a direct determinant of the decision to invest.

One major conclusion of our study is that crowdfunding platforms have the capacity to reflect the qualities of proposed start-up projects in a favorable manner to the entrepreneurs, having an essential contribution in influencing the factors presented above and in project financing, provided that they are used correctly and to their full potential. The reputation and type of platform, its audience, the type of crowdfunding campaign used, the duration of the campaign, the quality and quantity of information provided, the means of presenting such information, the language used, how frequently they are made available to the public, how campaign creators interact with the crowd of potential financers are all aspects that need to be studied in depth and fully understood, so that each of them can be set in accordance with the type of proposed project, its creator's intentions, as well as the expectations of the crowd of potential investors—and ultimately so that they can synergically result in accomplishing the desired financing goal. Furthermore, it is important to highlight that our study also revealed a necessity in terms of project planning: we believe that crowdfunding platforms should pay more attention to the progress of all the projects in the same category and dynamically decide the optimal moment to launch new projects, in order to reduce competition between projects that are close to reaching their funding goals, which significantly reduces the probability of being funded.

The practice showed that many start-ups that have no access to the other sources of funding resort are increasingly resorting to crowdfunding to obtain financing in their initial phase, as venture capital companies or funds reject the majority of proposals advanced to them and only invest in companies that could offer them the perspective of a high yield on the invested funds. Start-ups from different industries see crowdfunding as a viable alternative to traditional venture capital or initial public offerings, for the purpose of sourcing funds in a different manner, while also having a lower dilution of their own equity.

## Data Availability Statement

The original contributions presented in the study are included in the article/supplementary material, further inquiries can be directed to the corresponding author/s.

## Author Contributions

All authors listed have made a substantial, direct and intellectual contribution to the work, and approved it for publication.

## Conflict of Interest

The authors declare that the research was conducted in the absence of any commercial or financial relationships that could be construed as a potential conflict of interest.

## Footnotes

^1^Rayyan QCRI. https://rayyan.qcri.org/.
